# Anterior En Masse Retraction in Orthodontics

**DOI:** 10.7759/cureus.43194

**Published:** 2023-08-09

**Authors:** Kajal P Ahuja, Vikrant V Jadhav, Priyanka Paul, Hussain Ali John, Rishika Dakhale

**Affiliations:** 1 Orthodontics, Sharad Pawar Dental College and Hospital, Datta Meghe Institute of Higher Education and Research, Wardha, IND; 2 Orthodontics and Dentofacial Orthopaedics, Sharad Pawar Dental College and Hospital, Datta Meghe Institute of Higher Education and Research, Wardha, IND; 3 Public Health Dentistry, Sharad Pawar Dental College and Hospital, Datta Meghe Institute of Higher Education and Research, Wardha, IND; 4 Dentistry, Sharad Pawar Dental College and Hospital, Datta Meghe Institute of Higher Education and Research, Wardha, IND; 5 Oral Medicine and Radiology, Sharad Pawar Dental College and Hospital, Datta Meghe Institute of Higher Education and Research, Wardha, IND

**Keywords:** en masse retraction, orthodontic tooth movement, anterior retraction, orthodontics, sliding mechanics

## Abstract

This article reviews and critically analyzes the literature on mini-implants (temporary anchorage devices) for anterior en masse retraction in orthodontics. The search methods used were an E-database search, a secondary computerized search of orthodontics journals, and a reference list of selected studies. Eligibility criteria included individuals who underwent orthodontic treatment for correction of malocclusion with premolar extraction. Data were taken from PubMed and Scopus as well as the Cochrane Central Register of Controlled Trials and the Cochrane Database of Systematic Reviews. Keywords used for searching the article were temporary anchorage devices, premolar extraction, orthodontics, and anterior en masse retraction, Anterior en masse retraction with sliding mechanics in pre-adjusted edgewise appliances was considered for the study. Data collection and analysis involved three different researchers performing three steps of selection. All titles were initially filtered for irrelevant review articles. In the first step, all summaries from the selected studies were reviewed, and in the second, the entire content of the papers was read. The study was then discarded based on qualifying standards. A chart was created using the data from the final chosen research as well as the findings. The following information was evaluated for the final table: author, publication year, research structure, study group, sample size, methods/measures, study findings, and conclusion about frictionless mechanics. Results showed that a meta-analysis was not feasible due to clinical and statistical variability, as well as variations in study design, sample selection, and sample size. Thus, it was concluded that sliding mechanisms are widely employed in orthodontic treatment, but temporary anchorage devices and sliding mechanisms deliver great results. There is a need to raise awareness about these devices and use them with care.

## Introduction and background

Modern orthodontics emphasizes providing patients with the best care in the shortest amount of time. The length of orthodontic treatment can be impacted by a variety of variables, such as treatment plan, appliance type, mechanics, force, and patient-specific characteristics [1-3]. Reduced iatrogenic hazards, including root resorption and decalcification, benefit both patients and medical personnel by shortening treatment times [4-7]. Many products, including trans palatal arches, Nance arches, and headgear, are utilized to regulate anchorage since anchorage loss is a prevalent problem with orthodontic treatment. Extraoral appliances are thought to be unreliable and have been linked to incidences of facial damage [8,9]. They are helpful in anchoring control but depend on patient compliance. Orthodontic implants or temporary intraoral skeletal anchorage devices (TISADs) provide a compliance-free alternative to conventional anchorage [10].

Skeletal anchoring to retract upper anterior teeth is an old idea. Temporary anchoring devices (TADs) have been demonstrated to help with maxillary expansion, tooth intrusion, and space closure. Because mini-implants are adaptable, efficient, and simple to use, they have become increasingly important in the clinical management of orthodontic patients. According to TSADs, survival rates have ranged from 80% to 94% [11]. In order to achieve skeletal anchoring and avoid using the most popular intraoral and extraoral orthodontic equipment, which are typically unwanted to patients, various systems of mini-implants have been devised [12]. Comparing mini-implants to other less minimally invasive osseointegration systems for skeletal anchoring, they are now a safe, reliable, and effective technique [13]. The creation of mini-implants with high resistance and various shapes demonstrates that research on materials and miniaturization techniques is well-focused and has made significant progress, advancing implantology in the orthodontic sector [14,15].

In order to understand how patients feel about having braces, it is crucial to understand that their priorities can change depending on the type of treatment. Only a small number of studies have looked at anchoring supplements, with the bulk focusing on the pain or suffering associated with implant insertion or removal. In this study, individuals who needed en masse retraction of their anterior teeth were treated with TAD anchoring and standard maxillary bicuspid extractions.

## Review

Protocol and registration

This systematic review's protocol number was placed into the International Prospective Register of Systematic Reviews (PROSPERO) (CRD42023389228). The eligible studies for systematic reviews and meta-analyses were followed in this review.

Eligibility Criteria

Patients of any gender or race who were clinically undergoing premolar extraction and were systemically healthy (12 years or older) were included. Exclusion criteria include animal studies, case reports, literature reviews, lack of control groups, patients not receiving sliding mechanics, anchoring comparisons, and canine retraction loss. Interventions include* s*liding mechanics for anterior en masse retraction. This study aims to compare the different configurations of sliding mechanics used in treating patients with maximum anchorage. The outcomes of include the systematic study aimed to evaluate the time required for anterior en masse retraction, the amount of retraction achieved, the amount of anchorage loss, and the TAD failure rate during anterior en masse retraction. Research that was included had to be published in full journals, with the exception of enlarged abstracts of unpublished clinical research. Included are blinded outcome-assessment randomized controlled trials (RCTs). 

Sources of Data Collection and Study Selection

**Figure 1:**
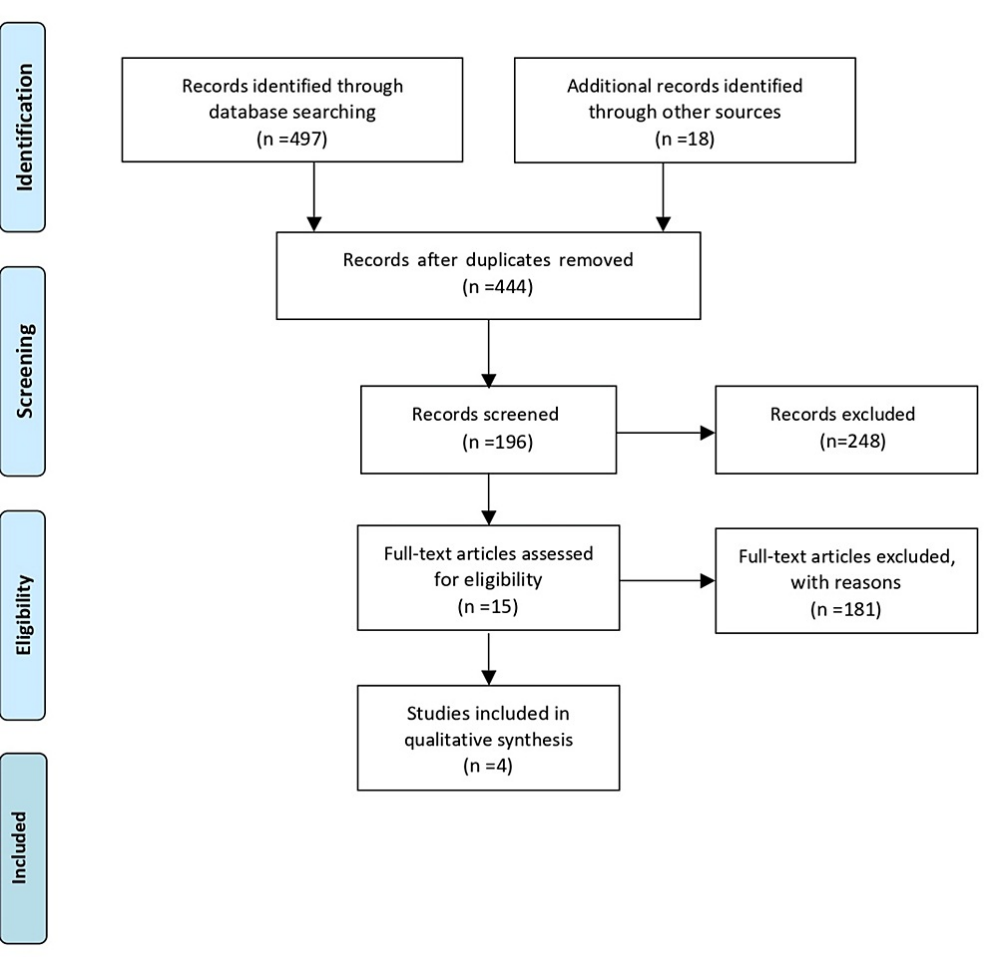
The selection process of articles used in study Adopted from Preferred Reporting Items for Systematic Review and Meta Analysis (PRISMA)

Up to 31 January 2023, unrestricted electronic literature searches were conducted. The Cochrane Collaboration provides access to database-specific limited data and terms to track databases: PubMed and Scopus, the Cochrane Central Register of Controlled Trials, and the Cochrane Database of Systematic Reviews. In addition, a comprehensive, thorough search of the bibliographies of all the selected articles was carried out Every effort was made to adhere to the Preferred Reporting Items for Systematic Reviews and Meta-Analyses (PRISMA) statement standards for transparent reporting of such studies. Figure 1 shows the selection of the study according to the PRISMA guidelines. Table 1 contains a precise evaluation of publications shortlisted for the present review. The following terms were used for the PubMed search for articles: "temporary anchorage devices", "orthodontics", "premolar extraction", "anchorage loss", and "anterior en masse retraction". Searching trial registries and grey literature databases yielded ongoing and unpublished investigations. Table 1 includes a list of included studies.

**Table 1:** Summary of characteristics of studies TADs: Temporary anchorage devices; TISADs: temporary intraoral skeletal anchorage devices

Author (year)	Study design	Study group	Sample size	Method used	Results of study	Conclusion
Barthélemi et al., 2019 [13].	Cross-sectional Survey	Patients aged 12 to 50 years	34	In order to retract the anterior teeth, patients will have bonding, bilateral extractions, and skeletal anchoring alignment. After levelling, four TADs will be inserted, and 100 g of loading force will be applied to the labial and buccal sides. The technique makes sure that the teeth are correctly aligned and retracted.	First maxillary molar movement is a secondary effect.	When appropriately implanted in the bone during the anterior tooth retraction phase, TADs are an effective anchorage source for maxillary anterior teeth, ensuring stability.
Sandler et al., 2014 [16].	Cross-sectional Survey	Patient aged 12-18 years needed maximum anchorage.	78	The McLaughlin, Bennett, Trevisi (MBT) 022 slot fixed mechanism was implanted in the patients.	No differences found in anchorage supplementation efficiency between headgear, Nance buttons, and TADs.	All three groups experienced the same level of success with anchorage support. More problems were produced by headgear and Nance buttons than by TADs, although TADs provided superior treatment. For individuals who require the most support, TADS may be the best option for bolstering orthodontic anchoring.
Sebastian et al., 2022 [15].	Randomized control trial	The kind of retraction subgroups were then subjected to subgroup analysis using parallel-arm and split-mouth investigations;		Cochrane Central, Ovid Medline, Embase, and other sources were used to find all pertinent studies.	A meta-analysis demonstrating a significant difference in tooth movement rate between nickel-titanium (NiTi) closed coil springs and elastomeric power chain was conducted. Also discovered to be more efficient than active ligatures are NiTi springs.	According to the NMA ranking, NiTi coil springs outperform active ligature with a 99% probability of closing voids. However, compared to elastomeric chains, their evidence is weaker.
Antoszewska-Smithet al., 2017 [14].	Cross -sectional		616 patients (451 female, 165 male)	Reinforced anchorage system in both maxilla and mandible with fixed mechanotherapy	According to a meta-analysis, utilizing TISADs enhances anchorage reinforcement over conventional methods. TISADs allowed for 1.86 mm more anchoring stability on average than conventional methods (P 0.001).	The meta-analysis's findings demonstrated that TISADs are superior to traditional anchoring reinforcement techniques. The typical difference of 2 mm appears to be clinically meaningful as well as statistically significant.

Data Extraction

Table 1 contains information that was taken from the three studies that the reviewers cited, including the kind of research, participant data, intervention specifics, and published findings. Disagreements will be settled by the fourth reviewer, and the difference in the risk of bias assessment will be settled by the fifth.

Risk of Blas (Quality) Assessment

Utilizing the Cochrane domain-based, two-part technique described in Chapter eight of the Cochrane Handbook for Systematic Reviews of Interventions (Higgins 2011), three reviewers will independently evaluate the risk of bias (RoB) of each included study. The fourth reviewer will settle the disagreement between the primary reviewers. The discrepancy in the risk of bias assessment will be resolved by the fifth reviewer. We will assess the RoB under the following domains such as sequence generation, allocation concealment, blinding, outcome evaluation, insufficient data, selective reporting, and other biases (Table 2).

**Table 2:** Risk of bias summary

Evaluation of the selected studies' quality				
Quality standards	Barthélemi et al., 2015 [13]	Sandler et al., 2014 [16]	Sebastian et al., 2022 [15]	Antoszewska-Smith et al., 2017 [14]
Random sequence generation	Yes	Yes	Yes	Yes
Allocation concealment	Yes	No	Yes	No
Blinding of participants and personnel	Yes	No	Yes	Yes
Blinding of outcome assessment	Yes	Yes	Yes	Yes
Income outcome data	Highly	Highly	Highly	Highly
Selective reporting	Yes	Yes	Yes	Yes
Other bias	Yes	Yes	Yes	Yes

Summary Measures and Approach to Data Synthesis

The predetermined findings of this review were the topic of a thorough qualitative synthesis [17]. We performed a pair-wise meta-analysis if the findings of two experiments on comparable subjects' treatments and outcomes were similar. This review's predetermined findings were the subject of a complete qualitative synthesis. We performed a pair-wise meta-analysis if the outcomes of the two trials' information on comparable treatments and outcomes were similar.

The I2 measure was used to quantify statistical heterogeneity, with arbitrary thresholds of 0- 40%, 30-60%, 50-90%, and 75-100% corresponding to non-significant, moderate, large, and considerable amounts of inconsistency, respectively. For insufficient information management, we attempted to get in touch with the actual author of the work if there was insufficient information so that we could get the crucial details that were lacking. The subject's treatment mechanics were investigated to identify clinical variation to avoid bias.

Result

The electronic database search yielded 515 items; however, after removing duplicate and irrelevant articles, only 444 remained. Following that, a final selection of 196 papers was based primarily on their whole texts, with just four research meeting our criteria. Four research studies were selected for our study which meet our criteria, according to the PRIMSA guidelines.

Study Characteristics

 Barthélemi's initial investigation looked at the efficiency of TADs during maxillary bicuspid extractions for individuals between the ages of 12 and 50. In the TAD group, TADs were employed, whereas the control group used conventional dental anchoring. Results revealed significant difference in TAD mobility, and TADs were identified as a trustworthy source of anchoring [13].

In their study, Sandler et al. examined the efficacy of headgear for anchorage supplements, Nance button palatal arches, and temporary anchoring devices in patients with malocclusions that required the most anchorage [16]. Using randomization in a 1.1.1 ratio, they conducted investigation on a total of 78 patients. During the time when anchoring supplementation was necessary, their principal result was a mesial molar movement. The secondary outcomes included dento-occlusal change, total treatment time, number of visits for care, casual and missed appointments, length of anchorage reinforcement, and patients' views of the anchorage supplementing technique. It was established that the three group's abilities to provide anchoring support were identical. TADs improved the treatment's quality. Therefore, TADs might be the best technique for strengthening orthodontic patients who require the greatest amount of anchoring.

The third study by Sebastian et al. compared various force delivery methods for closing orthodontic spaces using sliding mechanics [15]. According to the NMA rating, NiTi coil springs are the best method for space closure with a 99% likelihood. For the best outcomes, standardization of study designs and agreed-upon core outcome sets are required. In the fourth study, Antoszewska-Smith et al. examined the effectiveness of TISADs, also referred to as orthodontic miniscrew implants, in anchoring reinforcement during en masse retraction [14].

Individual Research Findings

Barthélemi concluded from the first study that TADs can be regarded as an effective source of anchoring during the retraction of maxillary anterior teeth. When TADs are properly implanted in the bone during the anterior tooth retraction phase, they remain stable [13]. According to the analysis of Sandler et al. (2014), there was no difference between the three group's capacities to offer anchoring support. Problems with nance buttons and headwear were more frequent than TAD problems. TADs raised the bar for medical care [16].

Sebastian's third piece from 2022 by using NiTi springs rather than an active ligature to close spaces, a meta-analysis found that the length of orthodontic treatment might be cut in half. However, it is possible that the difference between NiTi springs and elastomeric chains is not crucial from a therapeutic standpoint [15]. Antoszewska-Smith's fourth study from 2017 assesses the rate of mesial migration as its main finding [14]. Over the course of treatment, the success rate of mini-implants was seen. For both overall and subgroup studies, the mesial molar displacement between TADs and conventional anchors was compared, along with the mean difference and confidence interval. A supplementary outcome measure is the tip of the molars. The TADs were very successful [14].

Synthesis of Results

A meta-analysis was just not feasible because of clinical and statistical variability. Variations in study design, sample selection, and sample size restrict research comparisons even more.

Summary of Evidence

Given that four research studies met the prerequisites, it is important to carefully consider the current result. The paper that was selected is anterior en masse retraction in orthodontics. More scientific information on the clinical utility of sliding mechanics is needed, as evidenced by the lack of uniform investigations. In this double-blind, randomized clinical investigation, posterior anchorage outperformed traditional dental anchorage in terms of stability of the first maxillary molars during en masse retraction of the anterior teeth. Dental patient-reported outcome metrics were identified to evaluate the efficacy of care from the patient's viewpoint. A growing number of therapies have the potential to lessen patient suffering and enhance quality-of-life outcomes. In the current research, neither conventional nor micro-implant anchoring patients had any PROMs recorded. The patient's perceived advantage is therefore unclear. However, it is possible that since many conventional appliances, such as headgears, are extraoral, patients who wear the equipment can find it uncomfortable, which would result in lower levels of compliance than expected.

*Limitations* 

The articles that were chosen for the current systematic review on the system for anterior en masse retraction in orthodontics had a lot of limitations, to put it briefly. These shortcomings were mostly caused by the number and caliber of the papers that were reviewed. There is a need to raise knowledge about it since using sliding mechanisms along with temporary anchorage devices in a clinical setting demands increased professional expertise. More research utilizing more rigorous methodology is required to determine whether the general public is aware of the effectiveness of sliding mechanics in conjunction with TADs.

## Conclusions

The study discovered that using mini-implants for patients' maximum anchoring can lead to higher anchorage preservation than traditional techniques. By reducing the mesial rotation of the maxillary teeth and enhancing maxillary anterior segment retraction, mini implants lessen the anchoring loss. In some cases, the therapy time can be reduced by using TSADs. The typical difference of 2 mm appears to be clinically important as well as statistically significant. However, due to the average quality of the included studies, the results should be evaluated with care. More thorough research on this subject is required to allow for the development of more trustworthy judgments. This conclusion is in favor of using mini implants to ensure that patients are properly anchored.
